# Benign Paroxysmal Positional Vertigo Is Associated with an Increased Risk for Migraine Diagnosis: A Nationwide Population-Based Cohort Study

**DOI:** 10.3390/ijerph20043563

**Published:** 2023-02-17

**Authors:** I-An Shih, Chung-Y. Hsu, Tsai-Chung Li, Shuu-Jiun Wang

**Affiliations:** 1 Department of Public Health, College of Public Health, China Medical University, Taichung 404327, Taiwan; 2 Department of Neurology, Ching Chyuan Hospital, Taichung 428433, Taiwan; 3 Premium Healthcare Center, Chung Shan Medical University Hospital, Taichung 402306, Taiwan; 4 Graduate Institute of Biomedical Sciences, China Medical University, Taichung 406040, Taiwan; 5 Department of Healthcare Administration, College of Medical and Health Science, Asia University, Taichung 413305, Taiwan; 6 Department of Neurology, Neurological Institute, Taipei Veterans General Hospital, Taipei 11217, Taiwan; 7 Department of Neurology, National Yang-Ming Chiao Tung University School of Medicine, Taipei 11217, Taiwan; 8 Institute of Brain Science, National Yang-Ming Chiao Tung University School of Medicine, Taipei 11217, Taiwan; 9 Brain Research Center, National Yang-Ming Chiao Tung University School of Medicine, Taipei 11217, Taiwan

**Keywords:** benign paroxysmal positional vertigo, migraine, cohort study

## Abstract

Previous studies reported an increased risk of benign paroxysmal positional vertigo (BPPV) in patients with migraine. Hence, we aimed to assess the risk of migraine in patients with BPPV. This cohort study was conducted using the Taiwan National Health Insurance Research Database. The BPPV cohort consisted of patients aged <45 years with a diagnosis of BPPV between 2000 and 2009. An age- and sex-matched comparison group free from a history of BPPV or migraine was selected. All cases were followed up from 1 January 2000 to 31 December 2010 or until death or a diagnosis of migraine. The baseline demographic characteristics in both groups were compared using Student’s *t*-test and the chi-square test. Cox proportional hazards regression analysis was used to estimate the hazard ratio for migraine in the BPPV cohort compared with the comparison group after adjustment for age, sex, and comorbidities. Notably, 117 of the 1386 participants with BPPV and 146 of the 5544 participants without BPPV developed migraine. After adjustment for age, sex, and comorbidities, BPPV showed an adjusted hazard ratio indicating a 2.96-fold increased risk of migraine (95% confidence interval: 2.30–3.80, *p* < 0.001). We found that BPPV is associated with an increased risk of a migraine diagnosis.

## 1. Introduction

Benign paroxysmal positional vertigo (BPPV) is the most common cause of vertigo. A study reported that the lifetime prevalence rate of BPPV is 2.4% [1]. Idiopathic BPPV is more prevalent in older adults than in younger adults, with a peak onset between 50 and 60 years. It is also more prevalent in females than males, with a male-to-female ratio of 1:2–1:3. The typical symptom of BPPV is recurrent episodes of vertigo lasting for ≤1 min provoked by head movement. While each episode is brief, vertigo typically recurs in weeks without therapy [2] and is thought to be caused by the displacement of otoliths from otolithic organs into the semicircular canals [3,4]. However, the cause of the otolith displacement remains unknown. The Dix–Hallpike maneuver and head-roll test induce nystagmus in patients with suspected BPPV. After a clinical diagnosis of BPPV, Epley’s canalith-repositioning maneuver is used to treat patients immediately.

Migraine is the second most prevalent neurologic disorder, with a male-to-female ratio of 1:3 [5]. The 1-year prevalence rate of migraine was reported to be 9.1% in Taiwan [6]. The prevalence of migraine in males is highest in the age range of 25–29 years (8.3%), whereas it is highest in females in the age range of 30–34 years (21.1%). Most migraine diagnoses are made clinically according to the International Classification of Headache Disorders, 2nd Edition, 2004 [7]. With core symptoms and adequate follow-up time, a clinical diagnosis of migraine is primarily made in an outpatient setting. However, physicians should consider secondary headache disorder if the onset of migraine is noted after the age of 50 years [5,7]. Migraine is now a manageable disease entity with much evidence supporting preventive and abortive treatments [8]. Among the various subtypes of migraine, vestibular migraine introduced in the International Classification of Headache Disorders appendix, 3rd Edition, stands out with its presentation of vestibular symptoms lasting between 5 min and 72 h [9]. The duration of vestibular migraine symptoms is longer than that of BPPV [10,11]. Characteristic features of migrainous positional vertigo include short duration, frequent recurrence, onset in early life, migrainous symptoms during episodes of positional vertigo, and atypical positional nystagmus [11,12,13,14,15]. However, the vestibular migraine criteria are for research purposes, and better scientific evidence is warranted before vestibular migraine can be formally accepted.

As per Kayan et al., patients with migraine presented with vertigo three times more often than patients with tension-type headaches (26.5% vs. 7.8%) [16]. Dizziness and vertigo occur in 20–30% and 25–26% of patients with a primary complaint of migraine, respectively [17]. Hence, some clinical questions, such as “Do patients suffer BPPV before migraine onset?” and “If a patient had been diagnosed with BPPV, will the exposure to BPPV increase the risk of migraine in the patient?” arose. Even with multiple cross-sectional studies showing the association between migraine and BPPV, the main conundrum is that both disease entities are very frequent in the general population. However, there seems to be no cohort study investigating the incidence of migraine in adults with BPPV. Among the various types of vertigo and relatively nonspecific dizziness, we chose BPPV as the exposure in this cohort study because BPPV is the most common type of vertigo. This study aimed to investigate the risk of migraine in patients with BPPV using a retrospective cohort study design and a population-based research database.

## 2. Materials and Methods

### 2.1. National Health Insurance Research Database

The National Health Insurance Research Database (NHIRD) is a comprehensive database covering approximately 100% of the population in Taiwan. All information that would expose a person’s identity had been de-identified. The regulations from the Bureau of National Health Insurance (NHI) and the National Health Research Institute maintain the confidentiality of data. The 2000 Longitudinal Health Insurance Database (LHID) is a data subset randomly sampling 1 million people from the population between 1996 and 2010. The LHID used the International Classification of Disease, Ninth Revision, Clinical Modification (ICD-9-CM) to record disease entities. It is openly provided to researchers in Taiwan for scientific and epidemiological purposes. It includes the medical claims data for outpatient and inpatient services. The full ethical privacy of this study was approved by the Research Ethics Committee of China Medical University, and it was exempt from full review. This study used the outpatient records in the NHIRD as the data source.

### 2.2. Study Population

Given the background knowledge of the peak age of migraine onset being 25–35 years of age in Taiwan and the high possibility of misclassification bias in migraine diagnosis in patients >50 years, we collected data on newly diagnosed BPPV (ICD-9-CM code 386.11) cases between 2000 and 2009 from the LHID, with the baseline age being <45 years. First, patients with a previous diagnosis of migraine were excluded to avoid reverse causality. Then, we defined the index date of the BPPV case as the date of the initial BPPV diagnosis. Next, the date, month, and year of the index date of the selected case were assigned to the comparison cohort. Afterward, we adopted an individual matching method using age and sex at a ratio of 4:1 to randomly select individuals without a history of BPPV or migraine before the index date.

### 2.3. Baseline Characteristics

We included comorbidities before the index date as the baseline comorbidities, including hypertension (ICD-9-CM codes 401–405), diabetes mellitus (ICD-9-CM code 250), hyperlipidemia (ICD-9-CM code 272), anxiety (ICD-9-CM codes 300.0, 300.2, 300.3, 308.3, and 309.81), and depression (ICD-9-CM codes 296.2–296.3, 300.4 and 311), to further adjust for possible confounding factors. The comorbidities were selected because hypertension, diabetes mellitus, and hyperlipidemia are known risk factors for BPPV and BPPV recurrence [18,19], and anxiety and depression are known comorbidities in migraine [20].

### 2.4. Outcome

The primary outcome was the occurrence of migraine, defined as the first ambulatory visit associated with an ICD-9-CM code of 346, during the follow-up period. Both cohorts were followed up until 31 December 2010, or until death or the diagnosis of migraine.

### 2.5. Statistical Analysis

For descriptive statistical analysis, we used the chi-squared test and Student’s *t*-test to compare baseline demographic characteristics between the BPPV and matched control groups. We calculated the cumulative incidence of migraine for each group by dividing the total number of migraine events by the total sum of the follow-up person-years (per 10,000 person-years). We used the Cox proportional hazards regression model to analyze the overall, age-specific, sex-specific, and each comorbidity-specific risks of developing migraine associated with BPPV. Furthermore, we calculated the adjusted hazard ratio (aHR) of migraine presenting with a 95% confidence interval (CI) for the BPPV group after adjusting for age, sex, and comorbidities.

The statistical program SAS 9.3 (SAS Institute Inc., Cary, NC, USA) was used for all statistical analyses. The Kaplan-Meier estimate for measuring the cumulative incidence in both groups was calculated using R software (R Foundation for Statistical Computing, Vienna, Austria). Then, we compared the differences in the cumulative incidence curves between the two groups using the log-rank test. Statistical significance was set at *p* < 0.05 in a two-tailed test.

## 3. Results

### 3.1. Baseline Demographic Characteristics in the Study Groups

Within the 11-year study period, 1386 patients without previous a BPPV or migraine history who had a baseline age <45 years were diagnosed with BPPV. Based on individual matching using age and sex at a 4:1 ratio, 5544 patients were included in the control group after excluding those with a history of migraine or BPPV before the index date. The distribution of age and sex was similar between the two groups. In both cohorts, the mean age was 33.2 years. In addition, both cohorts had a female predominance (68.0%).

The baseline demographic status and comorbidities between the BPPV and control groups are shown in Table 1. The mean follow-up duration was 6.23 and 6.46 years in the BPPV and control groups, respectively. The prevalence rate of all comorbidities, including hypertension, diabetes mellitus, hyperlipidemia, anxiety, and depression, was considerably higher in the BPPV group than in the control group.

### 3.2. Comparison of the Incidence of Migraine Diagnosis between the BPPV and Control Groups

Table 2 shows the comparison of migraine incidence between BPPV and matched control patients. Within the 11-year follow-up period, 117 (1.35%) and 146 (0.41%) patients had migraine in the BPPV and control groups, respectively. The overall cumulative incidence of migraine was 3.31-fold higher in the BPPV group than in the matched control group (135.4 vs. 40.8 per 10,000 person-years). After adjusting for demographic characteristics, the BPPV cohort had a 2.96-fold higher risk of migraine than the matched control cohort (aHR: 2.96, 95% CI: 2.30–3.80).

**Figure 1:**
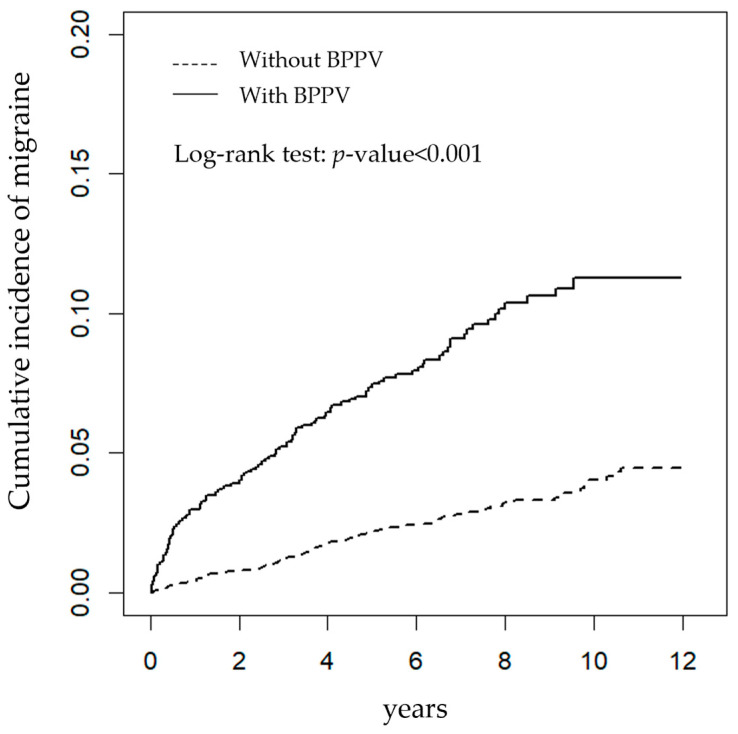
The cumulative incidence of newly diagnosed migraine using Kaplan-Meier survival analysis in patients with (solid line) and without (dashed line) benign paroxysmal positional vertigo (BPPV).

As shown in Figure 1, the BPPV group had a higher cumulative incidence of migraine than the matched control group, with the log-rank test showing the difference (*p* < 0.001).

### 3.3. Risk Factors for Migraine in Patients with BPPV

Using Cox proportional hazards regression analysis, a markedly higher hazard ratio of having a migraine in this specific BPPV sample and matched control cohorts was observed in females (aHR: 2.91; 95% CI: 2.30–3.80), those with hyperlipidemia (aHR:1.77; 95% CI: 1.16–2.70), and those with anxiety (aHR: 1.49; 95% CI: 1.03–2.14) (Table 3).

### 3.4. Subgroup Analysis of Migraine Risk

The subgroup analysis showed that an increased migraine risk was associated with age, sex, hypertension, diabetes mellitus, hyperlipidemia, anxiety, and depression (Table 4). However, sex may be associated with migraine occurrence in patients with BPPV. Male BPPV patients may have a more increased hazard ratio for developing migraine than female patients with BPPV. The migraine incidence in control males was 10.5 per 10,000 person-years, approximately one-fourth of the mean incidence (40.8 per 10,000 person-years) in the whole control group. This may amplify the increased aHR in the subgroup analysis of males compared with females.

### 3.5. Sensitivity Test

Further analysis of migraine risk stratified by different specialties diagnosing BPPV is presented in Table 5; this demonstrated a consistently higher migraine risk in the BPPV group stratified by different specialties making the BPPV diagnosis.

## 4. Discussion

This study mainly demonstrated that the risk of migraine was 2.96 times higher in the BPPV cohort than in the age- and sex-matched control cohort after adjusting for age, sex, and comorbidities. The incidence of migraine diagnosis was 135.4 per 10,000 person-years in patients with BPPV, whereas it was 40.8 in the age- and sex-matched control group. Furthermore, a higher risk of migraine was associated with three factors in patients aged <45 years and with a BPPV history: female sex (aHR: 2.91), hyperlipidemia (aHR: 1.77), and anxiety (aHR: 1.49). To our knowledge, this study is the first population-based cohort study to show that BPPV is associated with an increased risk of migraine.

Our study has various clinical implications. The atypical earlier onset age of BPPV among patients in clinical settings should prompt physicians to note migraine-related history, especially in female patients, those with a history of hyperlipidemia, or those with a history of anxiety. This is because these factors were independently associated with a higher migraine risk in patients with BPPV.

We limited the inclusion baseline age of patients to <45 years for three reasons. First, it avoids misclassification bias in the diagnosis of migraine in patients >50 years [10]. Second, the age specification limits the effect of baseline comorbidities and other unmeasured confounding factors leading to unmeasured selection bias and confounding regarding the risk of migraine between the two groups. Third, based on the 11-year timeframe of the database research and the peak age of migraine prevalence in males and females being 25–29 and 30–34 years, respectively, in Taiwan [6], it is reasonable to establish an age specification of <45 years to evaluate the age range associated with the highest risk as that between 25 and 34 years for the diagnosis of migraine in the included BPPV and age- and sex-matched control groups.

The mean baseline age of the BPPV and matched control cohorts were 33.2 and 33.1 years, respectively. However, this is not the typical prevalence age of patients with BPPV. Therefore, the number of patients in our BPPV group was relatively low. However, the mean baseline age is in the age range (25–34 years) of migraine peak prevalence in Taiwan. Furthermore, the male: female ratio was approximately 1:2, which is relatively consistent with the general epidemiological sex ratio of patients with BPPV. Comorbidities such as hypertension, diabetes mellitus, hyperlipidemia, anxiety, and depression were notably more common in the BPPV group than in the control group. While other methods, such as propensity score matching, may adjust the possible baseline demographic differences at the beginning of sample selection to reduce the selection bias and impact of confounding factors, the representative control group will also be changed from an age- and sex-matched general population sample to a more comorbidity-bound population sample. In this study, we aimed to compare the risk of migraine between patients with BPPV and a more general population to increase the external validity of the result.

Because our study showed a lower prevalence rate of migraine than the epidemiological study, we changed our paper title from “migraine” to “migraine diagnosis.” A previous study in Taiwan showed a 1-year migraine prevalence rate of 9.1% [6]. In our study, the incidence of migraine was 135.4 per 10,000 person-years in the BPPV group, whereas it was 40.8 in the age- and sex-matched control cohort. The 1-year prevalence rate of migraine in both cohorts was 1.35% and 0.41%, respectively, during the study period. This is far below the expected prevalence rate of migraine.

There are several epidemiological reports associating migraine with BPPV. However, to the best of our knowledge, there are neither cohort nor case-control studies investigating migraine risk in patients with BPPV. Nevertheless, a few cross-sectional studies have reported on the associations between migraine and BPPV. In a cross-sectional study by Lee et al., migraine is associated with BPPV with an odds ratio of 2.05 compared with the control group (*p* < 0.001) [21]. Another cross-sectional study showed that patients with BPPV had a three times higher historical rate of migraine than the general population and a higher family history of migraine (58.4% vs. 12.6%) and episodic vertigo (44.9% vs. 18%) in patients with BPPV than in patients with other dizziness and vertigo [22]. However, a cross-sectional study showed no significant association between migraine and BPPV in 508 patients in balance clinics [23]. Two population-based cohort studies investigated BPPV occurrence in patients with migraine. The first study, in Taiwan, showed a 2.03-fold higher risk of having BPPV in patients with migraine than in the age-, sex-, and comorbidities-matched controls (incidence rate ratio: 2.03; 95% CI: 1.41–2.97; *p* < 0.001) [24]. In the study with the same database as ours, no age specification was given due to the natural course of migraine peak age (25–34 years), followed by BPPV peak age (50–60 years). The second study, which had a larger sample size and a longer follow-up duration and was conducted in Korea, showed a 2.54-fold higher incidence of BPPV in migraine patients (aHR, 2.54; 95% CI:  2.41–2.68) [25]. The longer duration may more likely expose the patients in the migraine group between 50–60 years to BPPV. This may increase the likelihood of BPPV occurrence in the Korean study. Table 6 provides a general overview of the findings on the associations between BPPV and migraine in previous cohort studies.

Many observational surveys, including case series, have reported the association between BPPV and migraine [26,27,28,29,30,31]. The association between BPPV and migraine is not always consistent due to different study populations. Generally, these studies presented what is observed in the vertigo clinic, that patients with BPPV have a higher migraine prevalence. Patients with BPPV and a history of migraine tended to be younger with a female predominance. As presented by Faralli et al., the mean age was younger in patients with BPPV and migraine, and the recurrence rate of >4 documented episodes of BPPV was higher than in patients with BPPV without headache or migraine (39 vs. 53 years and 19.4% vs. 7.3%). No significant difference was observed in the number of maneuvers needed to achieve recovery between the two groups [27]. In headache clinics, neurologists seldom find patients with migraine with a history of BPPV [16,32]. Instead, in interviewing the patients, complaints of dizziness and vertigo existed. Therefore, observational reports suggested a higher prevalence of vertigo in patients with classical migraine than in patients with common migraine, tension-type headaches, and cluster headaches (42%, 12%, 17%, and 14%, respectively) [32]. Another study reported a higher vertigo prevalence in migraine than in tension-type headache (26.5% vs. 7.8% *p* < 0.001) [16]. Interestingly, there was no difference in neurotologic abnormality in patients with migraine presenting with or without vestibular symptoms [33]. As noticed in the literature, neurologists found a higher prevalence of vertigo in patients with migraine than in patients with tension-type headaches. Moreover, otolaryngologists found a higher migraine history in patients with BPPV than other dizziness and vertigo. The lack of time sequence between migraine and BPPV in cross-sectional studies and the lack of control groups in case series studies are the main limitations of the study design. A cohort study is the better observational study design in this situation. Another summary of published cross-sectional studies and case series studies investigating associations between BPPV and migraine with the exclusion of vestibular migraine is presented in Table 7.

The pathophysiological association between migraine and BPPV remains unclear. Three mechanisms associating migraine with BPPV have been previously proposed. First, vasospasm caused by migraine may be responsible for vertigo or cochlear symptoms. Baloh postulated that ontological symptoms in patients with migraine might occur from vasospasm or a certain iron channel disorder [34]. Ishiyama et al. proposed that repeated vasospasm can influence the microvasculature of the inner ear [35]. Repeated vasospasm may stress and damage the vestibular cells, thus resulting in the dislodgement of otoconia from the macula. The suppression of inner-ear microvasculature further leads to cochlear symptoms, such as hearing loss and vestibular symptoms. This association mechanism was supported by a previous study that showed an increased incidence rate of sudden sensorineural hearing loss in patients with migraine [36]. Second, the effect of neuropeptides, such as substance P, neurokinin A, nitrous oxide, and calcitonin gene-related peptide (CGRP), may cause sudden sensorineural hearing loss in patients with BPPV. CGRP is a neuropeptide involved in the efferent synapses of hair cell organs, such as the cochlea, semicircular canal, and lateral line [37]. Deletion of CGRP in transgenic mice was associated with reduced suprathreshold cochlear nerve activity and decreased vestibulo–ocular reflex gain. The recent success of many CGRP-related medications for migraine implies that the vestibular system plays an essential role in the pathogenesis of migraine [8]. Third, the CAMERA study demonstrated that a combination of hypoperfusion and embolism is the most likely mechanism for posterior circulation infarction in migraine [38]. The relative hypoperfusion may result in vertigo, as noted in vertebra–basilar insufficiency [38,39]. A cross-sectional study demonstrated the possible association between vertebral artery abnormality and BPPV [40].

Benign paroxysmal vertigo of childhood is one of the periodic childhood syndromes commonly regarded as a precursor of migraine, as described in the International Classification of Headache Disorders, 3rd Edition [9]. In this study, the annual conversion rate from BPPV to migraine was low (1.35%), with an 11-year accumulation incidence of 8.4%. In benign paroxysmal vertigo of childhood, the accumulation incidence rate of migraine with an average follow-up duration of 18 years and 15.7 years was 33.3% and 21%, respectively [41,42]. Hence, we were not able to infer further whether BPPV is a precursor to migraine. Rather, we postulated that a shared pathophysiological relationship might exist. This study’s result is also consistent with the previous notion that patients with acute migrainous vertigo develop central and peripheral vestibular dysfunctions [33].

This study has three major strengths. First, combined with the nationwide population-based cohort, the large sample size of one million citizens covered under the national health insurance in Taiwan and the 11-year adequate follow-up period for migraine occurrence provide enough power for differences between the two groups. Second, with the age specification of <45 years as the inclusion criterion, we tried to avoid a higher probability of migraine misclassification bias in the older population. Although some of the patients with migraine may present to the clinic after years of migraine suffering at age ≥50 years, we set the age specification not for definition purposes but rather for the need for validity. Moreover, with an age specification of <45 years in our study, the mean baseline age of both groups was 32 years. This was also good for migraine occurrence because migraine is a relatively early-onset disease entity with a peak onset age of 25–34 years in Taiwan. The 11-year follow-up period for migraine occurrence was adequate.

This study had some limitations. First, selection bias is prominent in our study, as shown in our baseline demographic state (Table 1), even with the baseline age specification of <45 years. Second, although known confounding factors, such as female sex, were controlled using a matching method, confounding is still a major source of bias in cohort studies because possible unknown confounders may exist. Third, misclassification of BPPV and migraine is still possible in this cohort study, even with the age specification to control the bias of inappropriate diagnosis of migraine in patients >50 years. However, it is worth noting that the differentiation of peripheral and central vertigo may occasionally be difficult in a clinical setting. In the case of BPPV, the difficulty may be ameliorated but not completely overcome. However, the lack of validation studies of BPPV in the database made this a serious limitation of our study. Finally, the severity, duration, and frequency of BPPV and migraine were not recorded in our database, and further exploration of their association was impossible.

## 5. Conclusions

Our study indicated a 2.96-fold higher risk of a migraine diagnosis in patients with BPPV than in age- and sex-matched controls in Taiwan. In addition, three other factors, including female sex, hyperlipidemia, and anxiety, were associated with an increased migraine diagnosis in this study.

## Figures and Tables

**Table 1:** Baseline demographic status of patients within the BPPV and control groups.

Characteristics		BPPV Group *n* = 1386	Control Group *n* = 5544	*p*-Value
		No. (%)	No. (%)	
Mean age, years (SD) *		33.2 (8.84)	33.1 (8.89)	0.74
Sex				>0.99
	Female	942 (68.0)	3768 (68.0)	
	Male	444 (32.0)	1776 (32.0)	
Comorbidity				
	Hypertension	134 (9.67)	221 (3.99)	<0.0001
	DM	44 (3.17)	83 (1.59)	<0.0001
	Hyperlipidemia	124 (8.95)	257 (4.64)	<0.0001
	Anxiety	234 (16.9)	292 (5.27)	<0.0001
	Depression	105 (7.58)	166 (2.99)	<0.0001

* Results are according to Student’s *t*-test. Abbreviations: BPPV: benign paroxysmal positional vertigo; DM: diabetes mellitus.

**Table 2:** Incidence of migraine in the BPPV and comparison groups using multivariate Cox proportional hazards regression analysis.

Characteristics	Patient with BPPV; *n* = 1386		Matched Cohort; *n* = 5544		Adjusted HR (95% CI)	*p*-Value
	Migraine, No.	Per 10,000 Person-Years	Migraine, No.	Per 10,000 Person-Years		
Total	117	135.4	146	40.8	2.96 (2.30–3.80)	<0.001

Model adjusted for age, sex, hypertension, diabetes mellitus, hyperlipidemia, anxiety, and depression. Abbreviations: BPPV: benign paroxysmal positional vertigo; HR: hazard ratio; CI: confidence interval.

**Table 3:** Incidence of migraine and multivariate Cox proportional hazards regression analysis measures of hazard ratio for the study cohort.

Variable	Event	PYs	Rate	Crude HR (95% CI)	Adjusted HR (95% CI)
**Age group**					
<20	15	4252	35.3	Ref	Ref
20–34	110	17,069	64.4	1.82 (1.06–3.12)	1.60 (0.93–2.76)
35–45	138	23,148	59.6	1.70 (0.99–2.89)	1.34 (0.78–2.31)
**Sex**					
Female	226	30,263	74.7	2.86 (2.02–4.05)	2.91 (2.05–4.13)
Male	37	14,207	26.0	Ref	Ref
**Hypertension**					
No	237	42,242	56.1	Ref	Ref
Yes	26	2228	116.7	2.09 (1.39–3.13)	1.50 (0.97–2.32)
**DM**					
No	259	43,611	59.4	Ref	Ref
Yes	4	858	46.6	0.79 (0.29–2.11)	0.47 (0.17–1.29)
**Hyperlipidemia**					
No	236	42,088	56.1	Ref	Ref
Yes	27	2381	113.4	2.01 (1.35–2.99)	1.77 (1.16–2.70)
**Anxiety**					
No	221	41,442	53.3	Ref	Ref
Yes	42	3028	138.7	2.54 (1.83–3.54)	1.49 (1.03–2.14)
**Depression**					
No	243	42,940	56.6	Ref	Ref
Yes	20	1529	130.8	2.25 (1.43–3.56)	1.30 (0.80–2.12)

Model adjusted for age, sex, hypertension, DM, hyperlipidemia, anxiety, and depression. Rate is the incidence rate per 10,000 person-years. Abbreviations: CI: confidence intervals; DM: diabetes mellitus; HR: hazard ratio; PYs: person-years.

**Table 4:** Subgroup analysis of migraine in the BPPV and age- and sex-matched control cohorts.

	Comparison Group			BPPV Group			Adjusted HR (95% CI)	*p* for Interaction
Comorbidity	Event	PYs	Rate	Event	PYs	Rate		
**Age group**								**0.68**
<20	9	3431	26.2	6	820	73.1	2.65 (0.92–7.66)	
20–34	62	13,749	45.1	48	3319	144.6	3.00 (2.03–4.44)	
≥35	75	18,645	40.2	63	4502	139.9	2.96 (2.09–4.20)	
**Sex**								**0.003**
Female	134	24,394	54.9	92	5868	156.8	2.50 (1.90–3.29)	
Male	12	11,432	10.5	25	2774	90.1	8.10 (3.99–16.4)	
**Hypertension**								**0.96**
No	137	34,412	39.8	100	7830	127.7	2.92 (2.24–3.81)	
Yes	9	1415	63.6	17	813	209.2	2.75 (1.18–6.42)	
**DM**								0.15
No	143	35,260	40.6	116	8352	138.9	3.01 (2.34–3.88)	
Yes	3	567	52.9	1	291	34.4	0.99 (0.10–9.79)	
**Hyperlipidemia**								**0.06**
No	131	34,244	38.3	105	7844	133.9	3.17 (2.43–4.13)	
Yes	15	1583	94.8	12	799	150.3	1.60 (0.74–3.45)	
**Anxiety**								**0.71**
No	135	34,170	39.5	86	7272	118.3	2.85 (2.16–3.75)	
Yes	11	1656	66.4	31	1371	226.1	3.67 (1.83–7.35)	
**Depression**								**0.84**
No	140	34,912	40.1	103	8028	128.3	2.88 (2.22–3.75)	
Yes	6	914	65.6	14	615	227.6	3.50 (1.31–9.39)	

Model adjusted for age, sex, hypertension, DM, hyperlipidemia, anxiety, and depression. Rate is the incidence rate per 10,000 person-years. Abbreviations: BPPV: benign paroxysmal positional vertigo; CI: confidence interval; DM: diabetes mellitus; HR: hazard ratio; PYs: person-years.

**Table 5:** Migraine risk stratified by different specialties diagnosing BPPV.

Variable	N	Event	PYs	Rate	Crude HR (95% CI)	Adjusted HR (95% CI)
Comparison cohort	5544	146	35,827	40.8	Ref	Ref
BPPV cohort						
Neurology	149	8	882	90.7	2.19 (1.08–4.47)	2.05 (1.00–4.19)
Otolaryngology	383	24	2175	110.3	2.65 (1.72–4.08)	2.49 (1.61–3.85)
Family medicine	214	18	1270	141.8	3.42 (2.09–5.58)	2.85 (1.73–4.69)
Internal medicine	310	33	2169	152.2	3.81 (2.61–5.57)	3.39 (2.30–5.00)
Others	203	18	1229	146.5	3.56 (2.18–5.81)	3.12 (1.90–5.12)

Model adjusted for age, sex, hypertension, diabetes mellitus, hyperlipidemia, anxiety, and depression. Rate: incidence rate per 10,000 person-years. Abbreviations: BPPV: benign paroxysmal positional vertigo; CI: confidence interval; HR: hazard ratio; PYs: person-years.

**Table 6:** Associations between BPPV and migraine in cohort studies.

Author Year Location Reference	Exposure Outcome	Matching Method	Matching Factors	Disease Coding	No. of Cases	Confounding Factors Adjusted	Brief Finding on Association
Chu 2015 Taiwan [24]	Migraine BPPV	1:1 PSM	PSM by age, sex, DM, HTN, heart failure, COPD, asthma, CKD, CAD, CVD, hyperlipidemia, cirrhosis, and autoimmune disease	ICD-9	8266 with migraine 8266 PSM controls	PSM	IRR of BPPV: 2.03 in migraine (95% CI: 1.41–2.97)
Kim 2019 Korea [25]	Migraine BPPV	1:4 individual matching	Age group, sex, income group, region of residence, and medical history of HTN, DM, and dyslipidemia	ICD-10	40,682 migraine, 162,728 controls	Matching factors and history of ischemic heart disease cerebral stroke, and depression	Migraine increased risk of BPPV (aHR: 2.54; 95% CI, 2.41–2.68)
Shih 2023 Taiwan (this study)	BPPV Migraine	1:4 individual matching	Age and sex	ICD-9	1386 with BPPV 5544 controls	Age, sex, HTN, DM, hyperlipidemia, anxiety, and depression	BPPV increased risk of migraine (aHR: 2.96, 95% CI: 2.30–3.80)

Abbreviations: aHR: adjusted hazard ratio; BPPV: benign paroxysmal positional vertigo; CI: confidence interval; CAD: coronary artery disease; CKD: chronic kidney disease; COPD: chronic obstructive pulmonary disease; CVD: cerebrovascular disease; DM: diabetes mellitus; HTN: hypertension; ICD-9: International Statistical Classification of Diseases and Related Health Problems 9th Revision; ICD-10: International Statistical Classification of Diseases and Related Health Problems 10th Revision; IRR: incidence rate ratio; PSM: propensity-score matching.

**Table 7:** Associations between BPPV and migraine in cross-sectional studies and case series studies.

Author Year Reference	Questionnaire Examination	Study Population	Major Finding
Uneri 2004 [22]	Motion sickness Migraine history FH of migraine FH of BPPV	477 BPPV 117 Control: other dizziness/vertigo	BPPV group vs. control group: FH of migraine: 58.4% vs. 12.6% FH of episodic vertigo: 44.9% vs. 18%
Pollak 2014 [26]	History of headache History of BPPV Background disease	53 headache with BPPV 52 age/sex-matched BPPV without headache	No difference in history of headache, history of BPPV, or background disease
Faralli 2014 [27]	Mean age Recurrence rate of BPPV	77 BPPV with migraine 109 BPPV without migraine or with other kinds of headache	Mean age in patients with BPPV and migraine compared with other kinds of headache (39 vs. 53 years) High recurrence rate of BPPV (>4 documented episodes (19.4% vs. 7.3%).
Yetiser 2015 [28]	Migraine	263 BPPV patients	32 patients had migraine (11.4%). Higher female: male ratio in BPPV with migraine vs. BPPV without migraine (4.2:1 vs. 1.3:1). No difference in mean age
Teixido 2017 [23]	Migraine	508 Balance clinic	No significant association between migraine and BPPV
Hilton 2020 [29]	Migraine	1481 BPPV patients	The prevalence of migraine among the study sample was 25.8%. Female gender, prior history of BPPV, younger age, and lack of diabetes were independently associated with the concurrent comorbidity of BPPV and migraine.
Lee 2021 [21]	BPPV and other vestibular symptoms	16,982 migraine 371,038 controls 103,699 non-migraine	Migraine: OR 2.05 for BPPV (*p* < 0.001). Non-migraine: OR 1.73 for BPPV (*p* < 0.001)
Grupta 2022 [30]	Migraine	100 BPV patients	34% with BPV with headache (including migraine), 10% with migraine
Kim 2022 [31]	Migraine	255 BPPV patients	44.7% had a history of migraine. Those with migraine had an earlier age of BPPV onset than individuals without migraine (60.2 vs. 65.4, *p* = 0.0018).

Abbreviations: BPPV: benign paroxysmal positional vertigo; BPV: benign positional vertigo; FH: family history; OR: odds ratio.

## Data Availability

The National Health Insurance Record Database is publicly available from the Taiwan National Health Institute. With academic or commercial requests, Taiwan citizens can have access to the research database.
